# Fabrication of PLLA/C_3_S Composite Membrane for the Prevention of Bone Cement Leakage

**DOI:** 10.3390/polym11121971

**Published:** 2019-11-30

**Authors:** Tsai-Hsueh Leu, Yang Wei, Yi-Shi Hwua, Xiao-Juan Huang, Jung-Tang Huang, Ren-Jei Chung

**Affiliations:** 1 Department of Mechanical Engineering, College of Mechanical & Electrical Engineering, National Taipei University of Technology (Taipei Tech), Taipei 10608, Taiwan; DAZ90@tpech.gov.tw; 2 Department of Orthopedics, Taipei City Hospital, Renai Branch, Taipei 10629, Taiwan; 3 Department of Chemical Engineering and Biotechnology, National Taipei University of Technology (Taipei Tech), Taipei 10608, Taiwan; wei38@mail.ntut.edu.tw (Y.W.); xj72baby@gmail.com (X.-J.H.); 4 Department of Medical Imaging and Radiological Sciences, Central Taiwan University of Science and Technology, Taichung 40601, Taiwan; yshwua@ctust.edu.tw

**Keywords:** kyphoplasty, polymethyl-methacrylate bone cement, barrier, poly-l-lactic acid, tricalcium silicate

## Abstract

Kyphoplasty is an important treatment for stabilizing spine fractures due to osteoporosis. However, leakage of polymethyl-methacrylate (PMMA) bone cement during this procedure into the spinal canal has been reported to cause many adverse effects. In this study, we prepared an implantable membrane to serve as a barrier that avoids PMMA cement leakage during kyphoplasty procedures through a hybrid composite made of poly-l-lactic acid (PLLA) and tricalcium silicate (C_3_S), with the addition of C_3_S into PLLA matrix, showing enhanced mechanical and anti-degradation properties while keeping good cytocompatibility when compared to PLLA alone and most importantly, when this material design was applied under standardized PMMA cement injection conditions, no posterior wall leakage was observed after the kyphoplasty procedure in pig lumbar vertebral bone models. Testing results assess its effectiveness for clinical practice.

## 1. Introduction

Osteoporosis is a bone disease with the symptoms of chronic pain in the waist or back, or neck pain due to a compression fracture, significantly affecting the life quality of the elderly [1,2]. To improve the cure rate and reduce low back pain and other symptoms of osteoporosis, percutaneous kyphoplasty (PKP) has been developed as a new treatment in which a gap was prepared in a fractured vertebral body using a balloon, followed by an injection of bone cement to restore the vertebral height [3,4].

Although kyphoplasty is an effective surgical treatment for an immediate reduction of pain and deformity that can accompany vertebral compression fractures, in certain cases, however, defects or clefts in the vertebral body may cause the cement leakage into the epidural space, paraspinal soft tissues, or disc space [5,6]. These cement materials, typically consisting of polymethyl methacrylate (PMMA) which offers mechanical stability, becomes a problem when the leakages of unreacted PMMA monomer liquid before cement polymerization in the bone bed occurred, which may cause remote organ embolism or local chemical or compress symptoms and even the thermal injury [7,8,9].

To avoid this problem, an eggshell technique was developed from Greene et al., to prevent the cement leakage with a small amount of cement applied as a protective eggshell injected firstly into the created cavity after balloon inflation during the kyphoplasty procedure [10]. Following which the balloon is re-inserted and inflated, creating a cement shell or a cement membrane around the inner walls of the created cavity, and another batch of cement was mixed and injected into the remaining cavity thereafter with limited cement leakage possibility from initial cement setting. Although this double cement application with PMMA as the cement anti-leakage shell or membrane has demonstrated its efficacy in clinical trial during the kyphoplasty procedure [11], the long term complications might be devastating due to PMMA presence on the bone cement surface [9], which makes the development of a new material design for anti-leakage membrane necessary. For example, Tetsushi Taguchi et al., had fabricated the reactive poly(vinyl alcohol) (PVA) membrane for the prevention of bone cement leakage with good potential for implantable balloon kyphoplasty, but further investigations on its clinical efficiency are still in progress [12].

On the other hand, polymeric membranes fabricated from a single material more often have limited biological performance compared to the use of hybrid biomaterials composed of biodegradable synthetic polymers and inorganic materials, with the hybrid biomaterials fitting better to bone tissue engineering applications due to their similar compositions to natural bones [13,14]. For example, biodegradable polymers such as poly(L-lactic acid) (PLLA) (L-lactic acid isomer of polylactic acid (PLA), an aliphatic thermoplastic polyester obtained by polymerizing lactide monomers) have been used for scaffold design doped with dicalcium silicate (C_2_S) nanoparticles as an ideal candidate for novel bone graft substitutes with enhanced mechanical and biodegradable properties, and biointeractive nature [15,16]. Scaffolds fabricated from (PLLA)/dicalcium phosphate dihydrate (DCPD) composite by indirect casting had also shown to effectively improve the mechanical strength and biocompatibility for the repair of bone defects when compared to the scaffold from neat PLLA [17]. From which the hybrid material may be a better candidate as the cement anti-leakage membrane design [17,18,19].

In this study, the hybrid membrane made of PLLA and tricalcium silicate (C_3_S) was designed to block cement egress during kyphoplasty procedures, with its optimized composition design, mechanical properties, biodegradability, biocompatibility, and anti-leakage capability investigated. This membrane could then find immediate use during kyphoplasty process where vertebral body defects may cause cement leakage.

## 2. Material and Methods

In this study, tricalcium silicate (C_3_S) powders prepared by a sol-gel process were added into poly(L-lactic acid) (PLLA) solution at different ratios to make the composite films using a solution casting method, with their mechanical properties including Young’s modulus, maximum stress, and fractural strain measured. Following which the membrane with optimized PLLA/C_3_S composition was characterized for its biodegradability, in vivo and in vitro biocompatibility and cytocompatibility, and in vitro anti-leakage capability.

### 2.1. Preparation of Tricalcium Silicate (C_3_S) Powders

Tricalcium silicate powders were synthesized by the sol-gel method, using Ca(NO_3_)_2_·4H_2_O and Si(OC_2_H_5_)_4_ (TEOS) from Showa, Tokyo, Japan, as the raw materials and nitric acid as a catalyst with an initial CaO/SiO_2_ molar ratio of 3. Briefly, 0.5 mol TEOS was added in 200 mL water under continuous stirring. The required amount of Ca(NO_3_)_2_·4H_2_O, as the calcium precursor, was added to the solution, and the solution was stirred for one hour. Then, the solution was maintained at 80 °C for 24 h until gelation occurred. The gel was dried at 120 °C and then calcined at different temperatures between 1300 and 1400 °C. The resultant powders were ground and sieved to 400-mesh for further characterizations.

The as-prepared Ca_3_SiO_5_ (C_3_S) powders were investigated by X-ray diffraction, XRD (X’Pert^3^ Powder, Malvern Panalytical LTD., Malvern, United Kingdom) and scanning electron microscopy, SEM (S-3000H, Hitachi, Berkshire, United Kingdom).

### 2.2. Fabrication of PLLA/C_3_S Composite Membrane

PLLA/C_3_S composite membranes used in this study were prepared by the solvent casting technique. The poly(L-lactide) (PLLA, inherent viscosity 0.55–0.75 dL/g in CHCl_3_) resin from Lactel Absorbable Polymers (Durect Corporation, Birmingham, AL, USA) was preconditioned in drying oven at 40 °C for 48 h to reduce the moisture content before use. The weight ratio of PLLA to C_3_S powders from the previous step was 100:1 (wt/%) in 30 mL of chloroform solution. The solution was sealed and stirred for 1 h at room temperature, following which the solution was cast onto a 120 mm × 20 mm (diameter × height) glass plate. The composite films were dried at room temperature in the venting hood for 4 h and then peeled from the glass plate within DI water for 10 min.

### 2.3. Characterization of PLLA/C_3_S Composite Membrane

#### 2.3.1. Tensile Tests

Mechanical properties of composite membranes were performed at room temperature using a universal tensile tester (CY-6102, Chun Yen Testing Machines Co., LTD., Taichung, Taiwan) at a crosshead speed of 10 mm/min. The Young’s modulus and maximum tensile strength of the PLLA/C_3_S composite membrane were determined from the linear region of stress-strain curves. Five samples of each composition were tested, and the average values ± standard deviation were reported.

#### 2.3.2. Morphological Features

The PLLA/C_3_S composite membrane was examined in an SEM (S-3000H, Hitachi, Berkshire, United Kingdom), with the morphology imaged at five keV after mounting and sputter coating with Au.

#### 2.3.3. Contact Angle Measurement

The apparent water-in-air contact angles of the PLLA/C_3_S films were measured by the sessile drop method using an FTA1000 goniometer (First Ten Angstroms, Inc., Portsmouth, VA, USA) at room temperature; 2 μL of deionized water (DI water) was dropped on the sample surfaces for the measurement.

#### 2.3.4. The Composite Membrane Porosity

The porosity of membranes under-analyzed was characterized using Mercury Porosimetry (Porous Materials Inc., Ithaca, NY, USA). Mercury porosimetry analysis is based on the intrusion of mercury into the porous membrane structure under controlled pressure applied. The pressure needed for intrusion according to the Washburn-equation [20].

(1) D=(1/P)×4γ × cosθ

Where D is the pore diameter, P is the applied pressure, γ is the surface tension of mercury, and θ is the contact angle between the mercury and the membrane sample. The volume of mercury penetrating the pores is measured directly at applied pressure. Membrane porosity refers to the void volume fraction of the membrane and defined as the volume of the pores divided by the total weight of the membrane in this study.

#### 2.3.5. Swelling and Degradation Studies

Swelling and degradation studies of the composite membrane were conducted in DI water under room temperature and phosphate-buffered saline (1× PBS, pH = 7.4, Sigma-Aldrich, Saint Louis, USA) under 37 °C, respectively. Briefly, 0.01 g of oven-dried material was weighed to obtain the dry weight (W_d_) and soaked thereafter in 1.0 mL of DI water or PBS for swelling and degradation tests, respectively.

For the swelling test, at each specific time point (i.e., 1, 7, 14 and 21 days), the membrane was removed, blotted gently with filter paper to remove surface water, and the swollen membrane was weighed again (W_w_), with the percentage of DI water absorbed by the membrane samples (% WA) calculated using the formula [21]:

(2) % WA=(Ww−Wd)/Wd × 100%

For the degradation test, the degradation percentage was calculated using the following formula [22]:

(3) % WL=(Wd−Wf)/Wd × 100%

Where W_f_ is the measured weight of the oven-dried membrane samples after soaking in 1.0 mL of PBS in thermostat shock sink at 37 °C, 50 rpm for different days (i.e., 1, 7, 14, 21, and 42 d). All experiments were done in triplicate, and the degradation results were presented as weight loss % (% WL) in the following sections.

#### 2.3.6. Thermal Properties

The glass transition temperature (Tg) and melting temperature (Tm) of the composite was determined by means of a differential scanning calorimeter (DSC 200, NETZSCH, Burlington, MA, US), fitted with a standard DSC cell, and equipped with a Discovery Refrigerated Cooling System (RCS90) (all TA Instruments, Delaware, USA). Samples of about 7 mg were placed into aluminum pans and subjected to two heating cycles from −90 °C to +110 °C with cooling and heating rates of 10 °C/min. The DSC cell was purged with dry nitrogen at 50 mL/min. The system was calibrated both in temperature and enthalpy with an Indium standard. Results are given as average value. All data were processed with Thermal Analysis software (TA Instruments, Delaware, USA).

### 2.4. Biocompatibility and Cytotoxicity of Membranes

#### 2.4.1. MTT Assay

In order to evaluate the cytotoxicity of composite membranes, extracts of the material and MTT (3-dimethylthiazol-2,5-diphenyltetrazolium bromide; Aldrich 135038, Sigma-Aldrich, Saint Louis, USA) assay were applied using L929 mouse fibroblasts as stated in the International Organization for Standardization 109993-5 [23,24]. Briefly, the extracts were prepared with sterile membrane samples incubated in the cell-cultured medium (1 g membrane in 5 mL of medium) at 37 °C under 5% CO_2_ atmosphere and extracted after 1, 7, 14, and 21 days. L929 fibroblasts were cultured in 24-well plates at a density of 5 × 10^4^ cell/well for 24 hr, with 1 mL of Dulbecco’s modified Eagle’s medium (DMEM, Gibco, Dublin, Ireland), containing 1% Non-essential amino acid (NEAA, Gibco), 1% Sodium pyruvate (SP, Gibco), 1% L-glutamine (L-G, Gibco), 1% prostate-specific antigen (PSA, Gibco) and 10% fetal bovine serum (FBS, Gibco). After that, the medium was replaced with 1, 7, 14, and 21-day extracts. After another 24 h, the extracts were removed, with 0.5 mL of 0.5 mg/mL MTT solution (0.2 mL of MTT mother solution (5 mg/mL of MTT in phosphate-buffered saline (1× PBS, pH = 7.4)) and 1.8 mL DMEM) added to each cell-containing well and incubated for about 4 h at 37 °C. Then, the MTT solution was removed carefully, and 1 mL of DMSO (D2650, Sigma, Saint Louis, USA) was added to each well to dissolve the formed formazan purple crystals. The formation of formazan product was analyzed by measuring absorbance at 570 nm using an ELISA microplate reader. The mitochondrial activity of the L929 fibroblasts was expressed as the result of cell viability (%): Relative absorbance from each condition over the control cultures (i.e., cells seeded on microplate and cultured in normal medium). Three replicates were analyzed for each sample, and the final data were expressed as the mean value ± the standard deviation.

#### 2.4.2. MG-63 Culture on Composite Membrane

Human MG-63 osteoblast-like cells were purchased from Bioresource Collection and Research Center (product No. BCRC 60279, passage 101, BCRC, Hsinchu City, Taiwan). A membrane sample such as PLLA or PLLA/C_3_S composite (10 mm × 10 mm) was placed in a 48-well plate (Thermo Fisher Scientific Inc., USA). After which, the MG-63 cells were seeded at a density of 5.0 × 10^4^ cells/well onto the plastic well of the plate in presence of the membrane and grow to confluence with 1 mL of DMEM (Gibco) containing 1% NEAA, 1% SP, 1% L-G, 1% PSA, and 10% FBS in each well. After 7, 14, and 7 days of culture, the medium in each well was removed and each well was rinsed with phosphate-buffered saline (1× PBS, pH = 7.4) three times with the remaining cells for the following investigations.

#### 2.4.3. Alkaline Phosphatase Activity 

ALP activity measurement was carried out to analyze the effect of phosphorylated chito-oligosaccharides on the osteoblast-like MG-63 cell line. Following the previous steps, the cells in each well were homogenized with 0.5 mL of 0.1% Triton-X100 in a sonicator for 5 min. The cellular activity was then measured by incubating for 60 min at 37 °C in 250 mM carbonate buffer containing 1.5 mM Magnesium chloride hexahydrate (MgCl_2_, Sigma, Saint Louis, USA) and 15 mM para-Nitro Phenyl Phosphate (p-NPP, J.T. Baker, Radnor, PA, USA). In the presence of ALP, p-NPP is transformed to p-nitro phenol and inorganic phosphate. The ALP activity was determined by measuring the absorbance at 405 nm in a spectrophotometer [25].

#### 2.4.4. In Vivo Animal Test and Surgical Procedures

**Figure 1:**
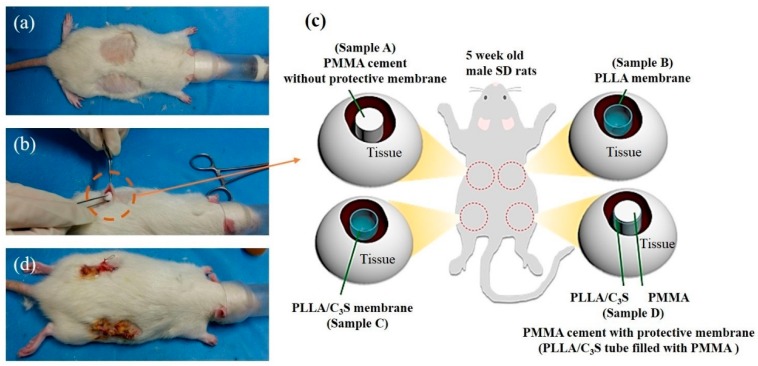
Implantation workflow. (**a**) Mice dorsal hair was removed and the skin was disinfected, (**b**) incision and the subcutaneous pocket on the rat back is formed with four different materials implanted with each of the material explained in (**c**), including polymethyl methacrylate (PMMA) (samples A), poly(L-lactic acid) (PLLA) membrane (sample B), PLLA/C_3_S membrane (sample C), and PLLA/C_3_S membrane tube filled with PMMA cement (sample D) prepared in a cylinder shape with the dimension of 20mm × 4.5mm (height × diameter). Sample A was considered as the positive control compared to other groups in this study. After which, (**d**) the incision was properly sutured and dressed.

In vivo animal studies were performed to assess the biocompatibility of the PLLA/C_3_S composites. All chemicals in this section were purchased from Sigma-Aldrich (Saint Louis, USA) and used without additional purification unless otherwise specified. All experimental animal protocols were carried out according to the Guide for the Care and Use of Laboratory Animals and approved by the National Taipei University of Technology, Taipei, Taiwan. All procedures were performed in Chang Gung Memorial Hospital with prior approval of the Institutional Animal Care and Use Committee (IACUC) of Chang Gung Memorial Hospital, Taoyuan City, Taiwan (2016112801). The animals used in this study were male Sprague-Dawley (SD) rats of 300 g body weight, on average, purchased from the National Laboratory Animal Center of Taiwan and acclimatized for a minimum of one week before experimentation. Animals had ad libitum access to standard rat chow and water at all times. All experimental procedures on given animals were completed under aseptic conditions. Each animal was anesthetized with 0.3 mL of a 1:2 mixture of Zoletil and Rompun. Once the rats were anesthetized, the dorsal hair was removed and cleaned using 70% volume fraction ethanol before the surgical procedures, as listed in Figure 1(a). Four horizontal incisions approximately 15 mm in length were made along each side on the back of the rat, and subcutaneous skin pockets were created by blunt dissection (Figure 1(b)). The pockets were separated by 40 mm to 50 mm. Each cylindrical-shaped sample (4.5 mm in diameter and 20 mm in length) was inserted into a pocket of subcutaneous tissues as shown in Figure 1(b), with 4 different samples (sample A (PMMA), B (PLLA), C (PLLA/C_3_S), and D (PLLA/C_3_S tube filled with PMMA)) implanted and located at different locations as shown in Figure 1(c). Subsequently, the incision was properly sutured, cleaned with 2% iodine, and dressed. For in vivo experiments, all implantations were one-shot treatments, with no replacement at any time point. The tissue specimens were obtained from four male SD rats after treatment for 7, 14, 21, and 28 days, with the portion of the selected tissue fixed with paraformaldehyde for 24 h at 4 °C, frozen and sectioned for hematoxylin-eosin (H&E) staining. The histological sections were examined using a stereomicroscope (Stereo Discovery, V20; Zeiss, Germany). All histological analyses were performed with at least three wounds per group per time point.

### 2.5. Anti-Leakage Tests of the Composite Membrane

The in vitro test of composite membrane for preventing bone cement leakage was conducted using fresh lumbar vertebral bones dissected from pigs and randomly divided into the blank group (without membranes), control group (with PLLA membrane), and the experimental group (with PLLA/C_3_S). The extra muscles, ligaments, and spinal marrow were eliminated and the vertebrae with the upper and lower endplates were completely retained. The models of vertebral compression fractures with fissures in anterior vertebrae were prepared by drilling two holes to a depth of 20 mm with a diameter of 4.5 mm. A small device called a balloon-tamp is covered with the composite membrane and then inserted through the needle and into the fractured vertebra. When the balloon-tamp is removed, it leaves a cavity covered with the membrane firstly and filled with the PMMA bone cement and then immediately sent for anti-leakage investigation using micro-computed tomography (Micro-CT).

All vertebrae samples were imaged using a benchtop Micro-CT imager (SkyScan 1076; Bruker-MicroCT, Kontich, Belgium) at 35 μm voxel image resolution with 100 kV, 100 μA, and a 1.0 mm aluminum filter.

### 2.6. Statistical Analysis

The results are expressed as the mean ± standard deviation (SD) with N ≧ 3. Data were analyzed by one-way ANOVA with the statistical function of Microsoft Excel.

## 3. Results and Discussions

In this context, we fabricated an implantable PLLA/C_3_S composite membrane that might be able to prevent the leakage of PMMA cement after kyphoplasty procedure, with its mechanical, in vivo, and in vitro biocompatibility, cytocompatibility, and in-vitro anti-leakage properties investigated.

### 3.1. Synthesis of Ca_3_SiO_5_ Powders

**Figure 2:**
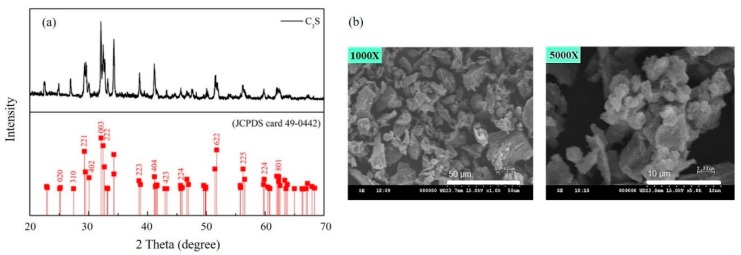
(**a**) XRD pattern and (**b**) SEM micrograph of prepared C_3_S powders.

PLLA/C_3_S composite membranes used in this study were prepared by the solvent casting technique, with tricalcium silicate powders synthesized by the sol-gel method [26] in our lab using Ca(NO_3_)_2_·4H_2_O and Si(OC_2_H_5_)_4_ (TEOS) as the raw materials. Figure 2(a) shows the XRD pattern of prepared Ca_3_SiO_5_ powder, also named C_3_S, with the results showing that it is triclinic and is the NO. 49-0442 in PDF standard card [27]. The grain size and surface morphologies of Ca_3_SiO_5_ powders were shown by the SEM micrographs (Figure 2(b)) which indicated that the Ca_3_SiO_5_ powders have a polygonal shape with the particle size of about 10–30 μm.

### 3.2. Characterization of PLLA/C_3_S Composite Membrane

The hybrid membrane made of PLLA and tricalcium silicate (C_3_S) was fabricated with its characterizations reported and discussed in the following sections.

#### 3.2.1. Tensile Properties

**Figure 3:**
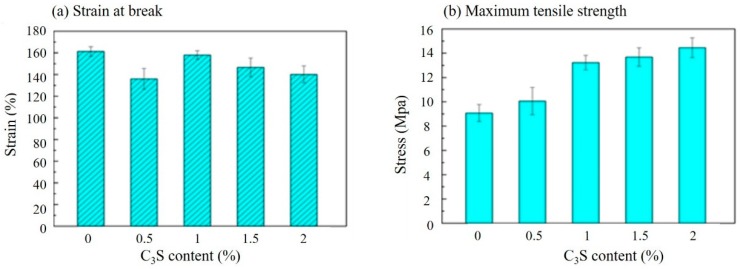
Tensile properties of PLLA/C_3_S composite membranes prepared at a various weight percent of C_3_S in PLLA (wt %) by (**a**) strain and (**b**) stress.

As shown in Figure 3, the strain at the break (Figure 3(a)) and maximum tensile stress (Figure 3(b)) of PLLA/C_3_S composite membrane was reported based on the varying weight percent of C_3_S in PLLA (i.e., 0%, 0.1%, 0.2%, 0.5%, 1%, 1.5%, and 2% (wt %). Accordingly, the maximum strength of the composite membrane increased with increasing C_3_S concentration, while the strain at break decreased when the C_3_S content is higher. The increased concentration of C_3_S might act as reinforcing filler for the increased bonding conditions of PLLA/C_3_S composite [28], which leads to its higher tensile strength and stronger resistance to the shape changes. From which the durability of the composite membrane, which shows the ability of the membrane specimen to resist changes of shape without cracking was decreased with the increasing concentration of C_3_S as well [29], which is in agreement with the general trend observed in Figure 3(a). However, our material design was aimed to cover the gap with the irregular shape prepared in a fractured vertebral body during the kyphoplasty in which case higher durability of the composite membrane is required. Therefore, to keep the relative higher tensile strength for a better barrier to prevent the cement egression while keeping higher plasticity or strain at break of our composite membrane design, 1% (wt %) of C_3_S in PLLA was selected accordingly as the optimized PLLA/C_3_S ratio for further membrane characterizations with the results discussed in the following sections.

#### 3.2.2. SEM Morphology and Surface Distribution of C_3_S within the Composite Membrane

**Figure 4:**
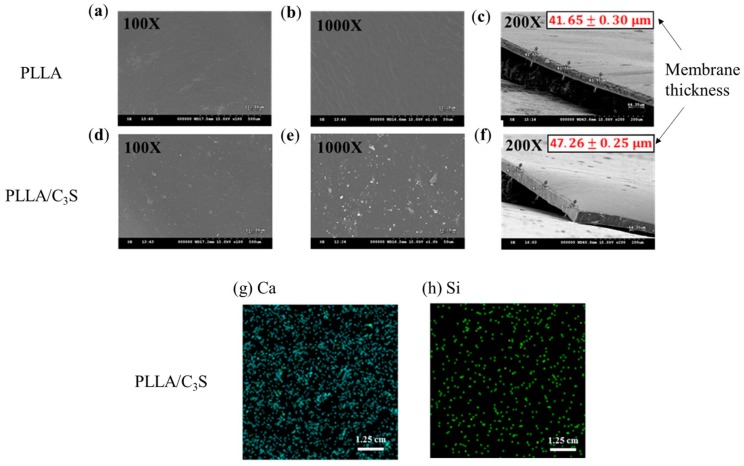
SEM micrograph of (**a**,**b**) prepared PLLA and (**d**,**e**) PLLA/C_3_S membranes. Membrane thickness was presented as mean ± SD with N = 3, as shown in the cross-sectional SEM micrograph of 4(**c**) and 4(**f**) for PLLA and PLLA/C_3_S membranes respectively. XRF mappings of chemical compositions of (**g**) Ca and (**h**) Si were shown within the prepared PLLA/C_3_S membranes.

Figure 4 showed SEM pictures of C_3_S particles precipitated in a PLLA membrane (1% (*w*/*w*)), with the membrane thickness presented from the cross-sectional SEM micrograph in Figure 4(c) and 4(f) for PLLA and PLLA/C_3_S membranes, respectively. The distribution of C_3_S within the membrane was further investigated using X-ray fluorescence (XRF) spectroscopy to characterize the chemical composition of Ca and Si on our composite membranes coated on a copper substrate. The precipitations of C_3_S on our membrane is significant for the properties of the hybrid biomaterials due to the enhanced binding affinity between the PLLA structures from the uniformly distributed Ca and Si within the membrane. As shown, the raw elemental maps for Ca and Si within the membrane were given in Figure 4(g) and 4(h), respectively, which indicated the uniform distribution of C_3_S content throughout the PLLA/C_3_S membrane.

#### 3.2.3. Contact Angle Measurement

**Figure 5:**
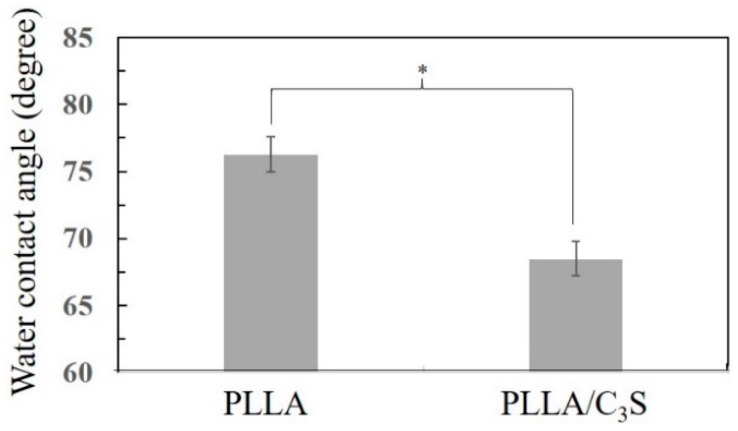
The water contact angle of PLLA and PLLA/C_3_S membrane, with data presented as mean ± SD for N > 3. (*P < 0.05).

Contact angle measurement is a standard method used to determine the hydrophilicity of a biomaterial surface. Figure 5 presents the contact angle values of PLLA and PLLA/C_3_S (i.e., 1% (*w*/*w*) of C_3_S in PLLA) composite determined by static contact angle measurement. Pure PLLA exhibits a contact angle of 76.3°, in agreement with the hydrophobic nature of the polymer [30]. With the addition of the C_3_S content (i.e., 1% (*w*/*w*) of C_3_S in PLLA), the contact angle decreases to 68.5° for PLLA/C_3_S composite, probably due to a reduction of interfacial tension between composite and water from the hydrophilic nature of C_3_S, making the PLLA/C_3_S composite surface more hydrophilic. Although the cell behaviors were found to be only partially influenced by the implanted biomaterial surfaces, cell spreading on the hydrophilic surface was usually more enhanced and uniformly spread compared to cells spreading on hydrophobic surfaces, due to a more complicated protein adsorption behavior involved such as unfolding on the hydrophobic substrate [31]. In which case PLLA/C_3_S could be a better-implanted material regarding biological applications when compared to the usage of neat PLLA polymer.

#### 3.2.4. Porosimetry Measurement

**Figure 6:**
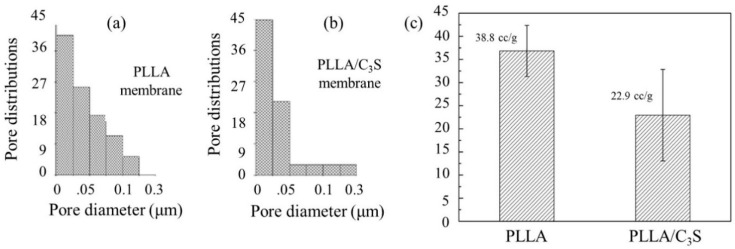
Porosity measurement of PLLA and PLLA/C_3_S membrane. Pore size and distributions of (**a**) PLLA and (**b**) PLLA/C_3_S. Total intrusion volumes of both membranes were presented in (**c**).

As shown in Figure 6(a) and (b), although both PLLA and PLLA/C_3_S (i.e., 1% (*w*/*w*) of C_3_S in PLLA) composite membrane have similar pore diameter in average, ranging from 20 to 30 nm, the broader size distribution of PLLA matrix was observed when compared to that of PLLA/C_3_S. This was further explained by the total intrusion volume data in Figure 6(c), also called the interstitial void volume (mL/g), representing the space between packed particles [32], with the interstitial void volume of PLLA matrix decreased from 38.8 to 22.9 (mL/g) after an addition of C_3_S (1% (*w*/*w*) of C_3_S in PLLA), which is also in agreement with our previous findings that C_3_S particles added to increase the binding conditions within PLLA matrix, which makes the tighter packing of PLLA chains as well as the narrower distributions of particle sizes.

#### 3.2.5. Swelling and Degradation Properties

**Figure 7:**
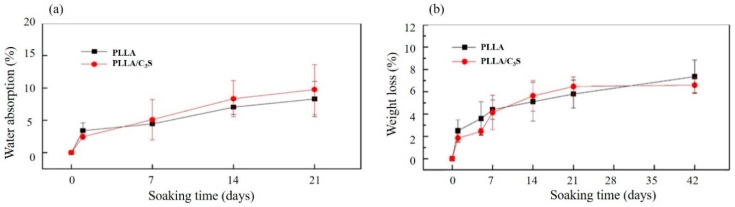
Swelling properties and degradation profiles of (**a**) PLLA and (**b**) PLLA/C_3_S membrane in DI water at room temperature and in phosphate-buffered saline (1× PBS, pH = 7.4) under 37 °C, respectively. Data represent mean ± SD with N = 3.

Poly(L-lactide) (PLLA) is more resistant to the degradation when compared to PLA alone due to its higher crystallinity, which makes the tensile strength of PLLA last longer [33]. The crystallinity of a polymer is also related to its swelling properties, which were primarily involved with the molecular chains in the amorphous region between lamella crystals, with more of these chains (e.g., higher amorphous contents or lower crystallinity) involved leading to a faster degradation rate due to the preferentially hydrolyzed properties of these chains [34,35]. As shown in Figure 7(a), the swelling index of PLLA has no significant difference when compared to that of PLLA/C_3_S composite, which suggests that the addition of C_3_S did not compromise the crystallinity of PLLA. This was further supported by the similar degradation profiles observed between PLLA and PLLA/C_3_S composite, as shown in Figure 7(b).

#### 3.2.6. Thermal Properties

**Figure 8:**
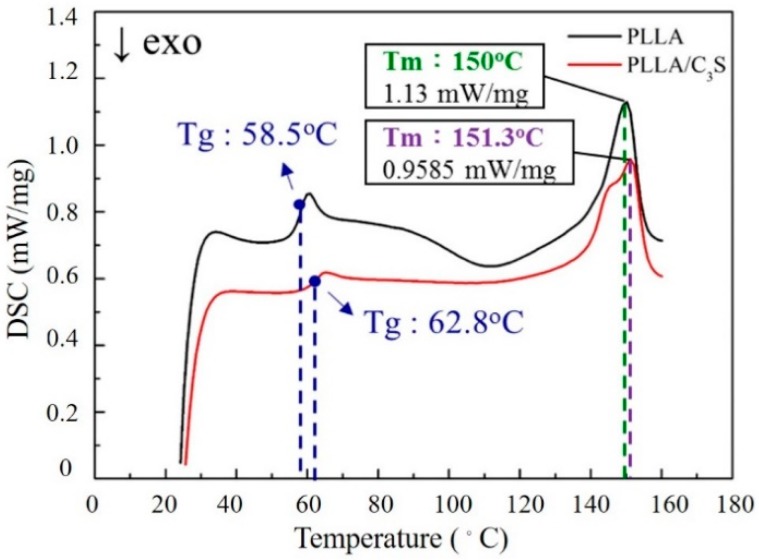
DSC scans of PLLA and PLLA/C_3_S composite melting thermograms.

DSC produces a heat flow (W/g) versus temperature (Celsius) curve, with the area under a melting transition curve of DSC trace representing the total amount of heat absorbed during the sample melting process, which would be proportional to the percentage crystalline by weight of the samples [36]. Figure 8 shows the melting endotherm for PLLA and PLLA/C_3_S composite samples during the heating process. As shown, these similar melting profiles suggest the crystallinity between PLLA and PLLA/C_3_S composite are not significantly different, which also confirms the intact PLLA crystallinity after the addition of 1% (*w*/*w*) of C_3_S.

### 3.3. Cellular Response

Cytocompatibility of PLLA and PLLA/C_3_S composite membranes were investigated using a MTT metabolic activity assay and the activity of alkaline phosphatase measurement, to observe the cell viability as well as cellular differentiation and proliferation when cultured with the PLLA and PLLA/C_3_S composite membranes.

#### 3.3.1. MTT Assay

**Figure 9:**
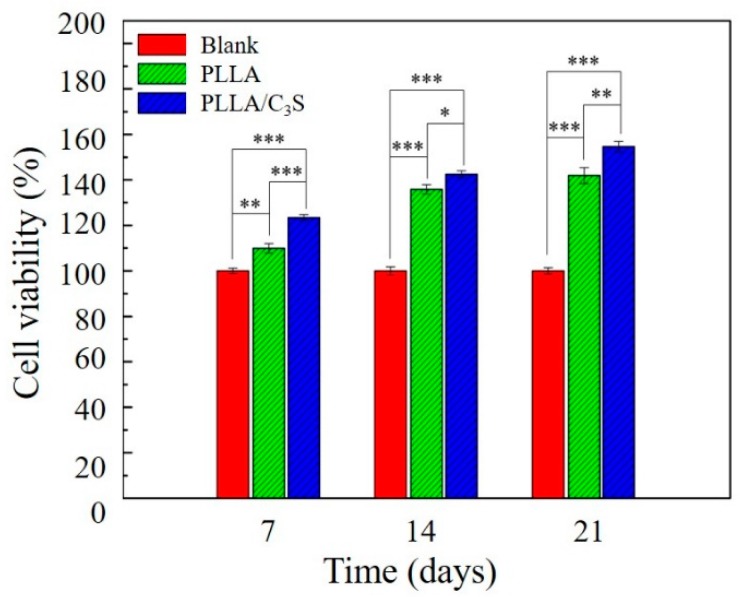
MTT cell viability measurement of L929 cells, which were seeded onto the surface of tissue culture plate (polystyrene) and cultured with PLLA or PLLA/C_3_S composite membranes. Cells grown within pure medium were used as blanks. An asterisk (*) indicates a statistically significant difference (p < 0.05); two asterisks (**) indicate a significant difference with p < 0.01, and three asterisks (***) indicate a significant difference with p < 0.001. No material interference. We had the membrane samples incubated in cell culture media without the cells, with the collected media showing no absorbance at 570 nm (data not shown), which confirms that nothing from the sample interfered with the absorbance at 570 nm.

This test was performed according to the International Organization for Standardization (ISO) norms [24], using the indirect contact method with extracts as applied to L929 cells, a line derived from mouse fibroblasts [23]. A MTT cell viability assay was performed to evaluate the effect of the C_3_S addition on the cellular performance of the PLLA/C_3_S composite samples in terms of cellular viability. As shown in Figure 9, the MTT results of the cytotoxicity levels of all sample extracts (i.e., blank group, PLLA, and PLLA/C_3_S composite) in direct contact with the L929s were given. As mentioned earlier, sample extracts were prepared with sterile samples such as membranes incubated in the cell-cultured medium (1 g sample in 5 mL of medium) and extracted after 7, 14, and 21 d. Cells grown within pure medium were used as blanks. Cell viability in these extracts was based on the mitochondrial enzymatic conversion of the tetrazolium into MTT formazan precipitated, occurring only in metabolically active cells. The amount of formazan dye directly correlates with the number of viable cells presented in the sample extracts [37]. The results indicated that the PLLA/C_3_S specimens had a significantly higher cell growth observed in comparison with other groups at each time points, with p < 0.05 indicated by one asterisks (*), p < 0.01 indicated by two asterisks (**) and p < 0.001 indicated by three asterisks (***). Results confirmed the lack of toxicity for all of the investigated samples.

#### 3.3.2. Alkaline Phosphatase (ALP) Activity Measurement 

Ceramic compositions have been shown to promote proliferation and differentiation of human osteoblasts into osteogenic lineages [38,39]. How the membrane material applied in this study will affect the cellular differentiation will be determined by ALP as an early marker of osteogenic differentiation, with the ALP playing a major role in the formation of mineral deposits in the matrix during new bone formation [40].

**Figure 10:**
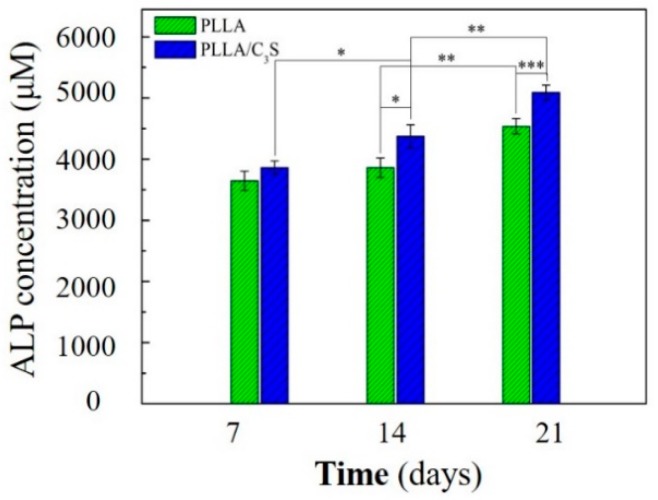
Cellular alkaline phosphatase (ALP) activity of MG-63 on PLLA and PLLA/C_3_S membranes. An asterisk (*) indicates a statistically significant difference (p < 0.05); two asterisks (**) indicate a significant difference with p < 0.01, and three asterisks (***) indicate a significant difference with p < 0.001.

Cell differentiation and proliferation of MG-63 induced by PLLA and PLLA/C_3_S membranes were evaluated by quantitative measurement of ALP activity. As shown in Figure 10, the ALP activity of the MG-63 cells increased in all groups for the incubation period of up to 21 days. On the 7th day, there were no significant difference observed in the ALP activity of the MG-63 cells cultivated on both membranes. On the 14th day, the ALP activity of MG-63 cells grown on PLLA/C_3_S increased significantly compared to that on PLLA (p < 0.05). On the 21^st^ day, more significant differences were observed in the ALP activity of MG-63 cells cultured on PLLA/C_3_S and PLLA membranes with p < 0.001, which indicates that PLLA/C_3_S could promote more of the differentiation of MG-63 cells. Therefore, the results of ALP activity showed the significance of C_3_S in cell differentiation, where the presence of C_3_S in PLLA might cause stimulation of bone cell response.

### 3.4. Qualitative Analysis of in Vivo Animal Tests

The purpose of this in vivo test was for the confirmation of the anti-leakages of PMMA cements from PLLA/C_3_S membrane and only the qualitative analysis of cellular reaction over time was studied. The PMMA cement, polymeric membranes (i.e., PLLA or PLLA/C_3_S membranes), and PMMA cement wrapped with PLLA/C_3_S membrane were investigated by implanting them in the subcutaneous pouch created on the dorsum of the Sprague-Dawley (SD) rats. Reentry of the implant site was done at different time points: Day 7, 14, 21 and 28 to assess the impact of polymers implanted on subcutaneous tissue, with the visual inspection showing that, the PMMA cement wrapped with PLLA/C_3_S membrane was appearing with the full integrity of the cavity in histologic specimens at all-time points which further suggest the anti-leakages of PMMA cements from PLLA/C_3_S membrane.

**Figure 11:**
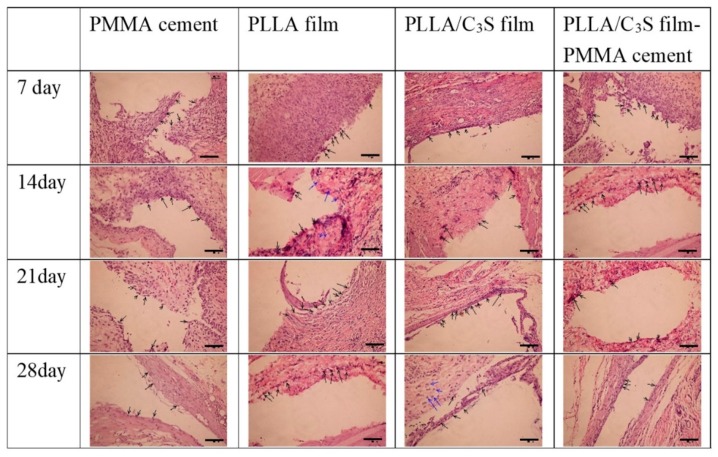
Histology of Sprague-Dawley (SD) rats skin. Each frame, from top to bottom, illustrates the histology of the tissues surrounding samples after different time points of subcutaneous implantation in a rat, with each sample appearing as the cavity surrounded by lymphocytes or macrophages, indicated by the black and blue arrows, respectively. (H&E x20; scale bar = 100 μm).

As shown in Figure 11, seven days after implantation, the membrane maintained its native structure and integrity and induced mononuclear cells, which were found on the membrane surface and the central region of each material was mostly free of cells. Although the presence of lymphocytes in histologic specimens of tissues surrounding implants have been present for several days, which might indicate that the inflammatory response to the implanted material is not over [41], the overall inflammatory response decreases after a longer implantation period. For example, after fourteen days following implantation, the numbers of lymphocytes and macrophages counted in averages/per section infiltrated at the site of implanted materials decreases, with more of the mononuclear cells invading the PLLA membrane observed when compared to that of PLLA/C_3_S implant. However, because the lymphocytes did not distribute homogenously, it might not be representative to present the average number of lymphocytes per section, and the quantitative histomorphometrical analysis of our samples is required for clinical practice in future research with the proper sampling method provided [42].

### 3.5. Anti-Leakage Tests

**Figure 12:**
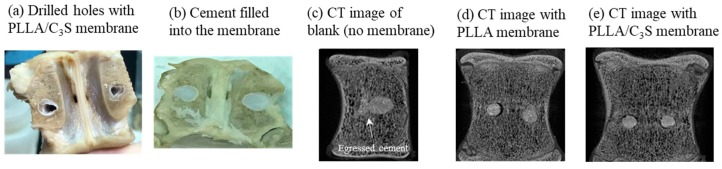
Anti-leakage investigation was carried out on simulated vertebral compression fractures by two drilled holes, in which the cavity within the pig vertebral bones was then filled with PMMA cement with or without membrane covered. (**a**) Photo of drilled holes with PLLA/C_3_S membrane; (**b**) photo of PMMA cement filled into the PLLA/C_3_S membrane; (**c**) micro-CT image without membrane (blank); (**d**) micro-CT image with PLLA membrane covered; (**e**) micro-CT image with PLLA/C_3_S membrane covered.

This final section was performed as the first investigation to assay the effects of PLLA/C_3_S composite on preventing cement leakage, with its in vitro observation of potential anti-leakage properties during the process of kyphoplasty to block cement egress through the posterior wall defect tested using fresh pig lumbar vertebral bones with holes drilled to represent the vertebral compression fractures and covered with the composite membrane using a balloon-tamp (Figure 12(a)). When the balloon-tamp is removed, it leaves a cavity with or without a PLLA or PLLA/C_3_S membrane-covered within the vertebral bones, which was then filled with PMMA bone cement soon after for anti-leakage investigation (Figure 12(b)). The photos and micro-computed tomography (Micro-CT) images are shown in Figure 12.

Our findings showed that, when the PLLA (Figure 12(d)) or PLLA/C_3_S composite (Figure 12(e)) membranes were applied during the kyphoplasty treatment before the PMMA cement injection, a better barrier outcome for the prevention of cement leakage was observed, especially when compared with the case without membrane used (blank), which has the significant amount of cement egressed through the posterior wall defect observed after the kyphoplasty (Figure 12(c)). Although both membranes, PLLA or PLLA/C_3_S, might be used as anti-cement leakage purpose, considering the lower tensile strength, bigger pore size, and more hydrophobic features of PLLA membrane, as discussed in our previous sections, the PLLA/C_3_S composite membrane should be a more reliable barrier with good potential to prevent the cement leakage in selected cases of vertebrae with posterior wall defects after the kyphoplasty procedure.

Of course, limitations of this experimental study at this stage merit to be mentioned. First of all, this is an in vitro study conducted in artificial vertebral analogs under selected environmental conditions (such as the fixed temperature and time of injections), which might be significantly varied in the clinical practice, with the cement leakage patterns much less predictable. Secondly, PMMA cement was the only cement tested using this anti-leakage membrane, which may not be applicable when other cements are applied. Many investigations have shown that cement leakage patterns in a vertebral body model may vary significantly especially when the viscosity of the cement was changed during the kyphoplasty procedures [43,44]. These weaknesses that may become obvious in clinical practice will be addressed in our continuous testing of PLLA/C_3_S composite membrane in cement augmentation techniques in selected cases of vertebrae with posterior wall defects. Finally, the original design of percutaneous kyphoplasty (PKP) has been developed as a treatment in which a gap was prepared in a fractured vertebral body using a balloon, followed by an injection of bone cement to restore the vertebral height, but the molecular mechanisms underlying such an effect long remained undetermined. For example, in previous histological observations, the PKP might evoke a local environment in the defect which might be conducive to the molecular mechanisms underlying coupled bone formation and bone remodeling during fracture healing via the recruitment and differentiation of multiple cell types, including osteoblasts and osteoclasts [45,46,47]. From this, the biological events in both the membrane and the underlying defect are important for bone regeneration and must be a key for a better design of this hybrid membrane made of PLLA and tricalcium silicate (C_3_S) to block cement egress during kyphoplasty procedures, which should be discussed in our future studies.

## 4. Conclusions

We have successfully created a potential solution for the posterior leakage during the kyphoplasty procedures using PLLA/C_3_S composite as anti-leakage membrane, with the addition of tricalcium silicate (C_3_S) into PLLA scaffold showing enhanced mechanical and anti-degradation properties while keeping good cytocompatibility, when compared to PLLA alone. Most importantly, when this material design was applied under standardized PMMA cement injection conditions, no posterior wall leakage was observed at the standard kyphoplasty procedure using pig lumbar vertebral bone as test models. In which case, the PLLA/C_3_S composite membrane might be a useful barrier material for implantable balloon kyphoplasty.
